# Acute malnutrition recovery energy requirements based on mid-upper arm circumference: Secondary analysis of feeding program data from 5 countries, Combined Protocol for Acute Malnutrition Study (ComPAS) Stage 1

**DOI:** 10.1371/journal.pone.0230452

**Published:** 2020-06-03

**Authors:** Rachel P. Chase, Marko Kerac, Angeline Grant, Mark Manary, André Briend, Charles Opondo, Jeanette Bailey

**Affiliations:** 1 Department of International Health, Bloomberg School of Public Health, Johns Hopkins University, Baltimore, Maryland, United States of America; 2 Department of Population Health & Centre for Maternal, Adolescent and Child Health (MARCH), London School of Hygiene and Tropical Medicine, London, England, United Kingdom; 3 Action Against Hunger-USA, New York, New York, United States of America; 4 Department of Pediatrics, Washington University in St. Louis School of Medicine, St. Louis, Missouri, United States of America; 5 University of Tampere, University of Tampere School of Medicine, Center for Child Health Research, Tampere, Finland; 6 Department of Nutrition, Exercise and Sports, University of Copenhagen, Copenhagen, Denmark; 7 Department of Medical Statistics, London School of Hygiene and Tropical Medicine, London, England, United Kingdom; 8 International Rescue Committee, New York, New York, United States of America; Addis Ababa University School of Public Health, ETHIOPIA

## Abstract

**Background:**

Severe and moderate acute malnutrition (SAM and MAM) are currently treated with different food products in separate treatment programs. The development of a unified and simplified treatment protocol using a single food product aims to increase treatment program efficiency and effectiveness. This study, the first stage of the ComPAS trial, sought to assess rate of growth and energy requirements among children recovering from acute malnutrition in order to design a simplified, MUAC-based dosage protocol.

**Methods:**

We obtained secondary data from patient cards of children aged 6–59 months recovering from SAM in outpatient therapeutic feeding programs (TFPs) and from MAM in supplementary feeding programs (SFPs) in five countries in Africa and Asia. We used local polynomial smoothing to assess changes in MUAC and proportional weight gain between clinic visits and assessed their normalized differences for a non-zero linear trend. We estimated energy needs to meet or exceed the growth observed in 95% of visits.

**Results:**

This analysis used data from 5518 patients representing 33942 visits. Growth trends in MUAC and proportional weight gain were not significantly different, each lower at higher MUAC values: MUAC growth averaged 2mm/week at lower MUACs (100 to <110mm) and 1mm/week at higher MUACs (120mm to <125mm); and proportional weight gain declined from 3.9g/kg/day to 2.4g/kg/day across the same MUAC values. In 95% of visits by children with a MUAC 100mm to <125mm who were successfully treated, energy needs could be met or exceeded with 1,000 kilocalories a day.

**Conclusion:**

Two 92g sachets of Ready-to-Use Therapeutic Food (RUTF) (1,000kcal total) is proposed to meet the estimated total energy requirements of children with a MUAC 100mm to <115mm, and one 92g sachet of RUTF (500kcal) is proposed to meet half the energy requirements of children with a MUAC of 115 to <125mm. A simplified, combined protocol may enable a more holistic continuum of care, potentially contributing to increased coverage for children suffering from acute malnutrition.

## Introduction

Acute malnutrition (AM) is divided into Severe Acute Malnutrition (SAM) and Moderate Acute Malnutrition (MAM), with SAM defined as a MUAC <115mm, weight-for-height z-scores below -3 of the median WHO growth standards or by the presence of nutritional oedema, and MAM defined as MUAC 115 to <125mm or weight-for-height z-scores of between <-2 and ≥-3. Globally, at any one time, it is estimated that more than 50 million children under the age of five suffer from acute malnutrition (AM) [1], likely translating to over 100 million incident cases of SAM each year [2–5]. SAM accounts for approximately 516,000 deaths annually, MAM for 359,000 [6,7].

Currently, SAM and MAM are treated in separate feeding programs, with separate protocols, products, and supply chains [8]. Resource constraints and logistical challenges often result in SAM being prioritized due to its particularly high case-fatality and long-term adverse outcomes [9,10]. MAM services are often unavailable or time-bound (e.g. only present during the ‘hungry’ season or in times of food crisis). Increasing evidence suggests that this is an important gap: children with MAM-associated anthropometric deficits (a weight-for-height z-score between -3 and <-2) are 3 times more likely to die than healthy children [11], and prevalence of MAM can affect the incidence and severity of SAM [12,13].

To better tackle both MAM and SAM, there is growing momentum within the nutrition community to view the two as a continuum condition rather than as two distinct states [14–17]. Thus simplifying and unifying the treatment protocols might ease integration into existing health services, increase treatment coverage, and potentially enhance cost-effectiveness by treating MAM earlier and more easily, preventing costly and dangerous SAM [15,16,18]. Early data on such programming is promising [18]. However, to inform future policy decisions, stronger evidence is needed [19].

Two questions are key. First, can mid-upper arm circumference (MUAC) be used as the primary anthropometric indicator for screening, treatment, and discharge of uncomplicated cases of acute malnutrition? MUAC-only programming offers many practical and logistical advantages over weight-for-height [20]. MUAC for admission has been found to detect the highest-risk children [21,22]. However, further evidence is needed on MUAC as a patient monitoring and discharge criterion. In particular, does MUAC reflect similar trends as weight gain in response to nutritional treatment [23–25]?

Second, how much energy is needed to most efficiently treat acute malnutrition? A wide variety of products and approaches are available for addressing MAM, not only limited to treatment [14,26,27]. Studies show that nutrient-dense pastes (Ready-to-Use Therapeutic Food, or RUTF) that are typically recommended for treatment of SAM result in higher recovery rates for MAM patients than do fortified blended flours [28–34]. For SAM, RUTF is standard and is prescribed to provide 175–200 kcal/kg/day [35,36]. This energy dosage is high because it is based on inpatient-focused models of care where expected patient weight gain was high: 10–15 g/kg/day [37]. In contrast, expected weight gain in today’s outpatient-focused programmes is about 4.5 to 6.8 g/kg/day [38]. Children may not thus require all the energy they are prescribed, especially as growth slows towards the end of treatment [39–42].

We aimed to address these evidence gaps by assessing MUAC and proportional weight gain, and estimating energy needs of children as they recover from SAM and MAM. The overall aim of this ComPAS Stage 1 study was to design a simplified, MUAC-based dosage protocol and statistically assess its theoretical performance providing adequate energy. Driven initially by an immediate need to inform a cluster-randomised control trial on a “Combined Protocol for Acute Malnutrition Study” (ComPAS study) [43], our goal has been to inform and ultimately improve wider SAM/MAM programming. Specific objectives are threefold. First, we aim to describe and compare MUAC and weight gain trends in children being treated for acute malnutrition to assess if MUAC is a valid proxy of proportional weight gain during treatment. Second, we will calculate energy requirements needed to support observed weight and MUAC gain in children recovering from acute malnutrition and to explore any differences by age and by MUAC category (SAM vs MAM). Third, we will assess the ability of a simplified MUAC-based dosage protocol to provide energy sufficient to meet estimated needs of children recovering from acute malnutrition.

## Methods

### Study design

This study was a secondary analysis of routine clinical data obtained from therapeutic and supplementary feeding programs (TFP and SFP, respectively).

### Setting and study sample

We analyzed data from programmes run by Médecins Sans Frontières (MSF-France) in South Sudan in 2010; Action Against Hunger (AAH-USA) in Pakistan in 2012; and International Rescue Committee (IRC) in Chad (2013 & 2014), Kenya (2012, 2013, & 2014), and Yemen (2014) as these programs had treatment data (rather than survey or cross-sectional data) and a partnership to do this research. Admission of children to these outpatient TFPs for SAM or SFPs for MAM were based on screenings in the community or directly at the health centre. Admission criteria for both TFPs and SFPs followed standard international criteria, as laid out in national acute malnutrition treatment protocols in use at the time in that particular country [44–47]. Enrolled patients had to be clinically well, alert and demonstrate appetite. Acute malnutrition cases eligible for treatment were enrolled in the TFP or SFP programme and monitored on a weekly basis (SAM) or a two-week basis (MAM). Therapeutic rations for SAM patients were provided based on weight (200 kcal/kg/day) and supplementary rations for MAM patients were provided as a standard ration which varied by country. Patients were discharged when they reached a combination of discharge criteria, which differed between TFP and SFP and also varied by country.

Patient data were originally available on paper-based patient monitoring cards held securely on-site at each programme. Data from the cards were entered into an Excel database between October 2014 and May 2015 using double data entry for quality control. The main study team at IRC received anonymized data and merged it into a single database stored on a password-protected institutional server. We tagged data by type of facility (TFP or SFP) and country from which the cards were obtained.

Participating children were those aged 6 to 59 months and enrolled in either outpatient TFPs for SAM or SFPs for MAM. Children were eligible for analysis if they were discharged from a feeding programme as recovered, experienced a non-negative MUAC gain, and experienced either a weight gain of at least 10% from admission to discharge if admitted as a SAM patient or at least 3% from admission to discharge if admitted as a MAM patient. By using data from patients who achieved these outcomes, trends reflect the most successful cases coming out of current TFP and SFP programs. We chose to focus on children who had achieved recovery in order to establish energy requirements for successful growth.

Exclusion criteria were those children with unusable data, defined as having no follow-up visits, or missing age, sex or date information. Data analysis was limited to MUACs 100mm to 140mm due to limited information outside these values for reliable estimates of weight change, MUAC change, and energy needs within each MUAC category.

Visits recording extreme high or low anthropometric measures which are thus likely to be measurement or recording errors were also excluded according to the following criteria [48]: weight-for-height z-score below -5 or above 5, weight-for-age z-score below -6 or above 5, height-for-age z-score below -6 or above 6, MUAC below 65mm or above 200mm, weight change of more than 25 grams per kilogram weight per day, or MUAC change in either direction of greater than 15mm per week.

### Variables, measurements, and statistical methods

Variables available on the patient cards included: date of visit to clinic, age in months at admission, presence of oedema at admission, mid-upper arm circumference (MUAC) measured in millimeters (mm) at each visit, weight measured to the tenth of a kilogram (kg) at each visit, and height measured to the tenth of a centimeter (cm) at admission and discharge. Each card followed an individual child through one course of treatment. Not all children completed treatment, and registration cards could not necessarily be linked if the same child had multiple courses of treatment or obtained treatment for both SAM and MAM in separate programmes.

The total number of study participants was determined by available programme data. Because this was a secondary analysis, *a priori* sample size calculations were not done. Five different countries were included so as to represent a variety of different settings where acute malnutrition is highly prevalent.

From the total eligible sample, we used different subsamples for analyses. For polynomial smoothing used to visually compare trends in weight and MUAC change since prior visit by MUAC at prior visit, we randomly selected 1000 visits from each of the five countries from which patient cards were obtained, resulting in a single 5000-visit subsample with an equal number of observations from each country (to which Yemen could contribute the least with 1279 eligible observations). One-hundred such subsamples were used to test whether the linear trend of the difference in the normalized trends was non-zero. For theoretical assessment of the proposed therapeutic protocol, we used 100 different subsamples of 2500 visits (500 visits from each country) to simulate average performance of the protocol given varying distributions of patient visits from the sample. To estimate indicators to two significant figures, 100 resamples were sufficient.

We conducted the analysis using Stata 13.1 [49]. Weight-for-height (WHZ), weight-for-age (WAZ) and height-for-age (HAZ) z-scores were calculated at admission and discharge using the 2006 WHO child growth standards via the user-written Stata command zscore06 [50]. One-week change in MUAC was calculated as the difference between MUAC between two visits (usually one week apart). If visits were not one week apart, change was assumed constant over the period of time between visits, and so change over the course of two weeks was divided in half to reflect one-week change in measurements. Proportional weight gain was similarly assumed constant between visits, and was calculated as grams of weight gained per kilogram of weight at prior visit per day. Change in MUAC (mm/week) and proportional weight change (g/kg/day) were primary outcomes with MUAC the predictor.

Using local polynomial smoothing (via the lpoly command) with the 5000-visit subsample, we visually assessed one-week MUAC growth (mm/week) versus MUAC and proportional weight gain (g/kg/day) versus MUAC. This was done among all patients with eligible outcomes to understand whether MUAC and weight change responded similarly to treatment. A secondary analysis was performed assessing these by age group to determine if the relationships were similar for 6–11 month olds, 12–23 month olds, and 24–59 month olds. A set of 100 simulations (each with a different 5000-visit subsample as used for local polynomial smoothing) assessed via linear regression whether the difference in normalized means of MUAC growth and proportional weight gain had a non-zero trend.

Daily energy needs (kcal) were calculated as [51,52]:

(currentweight(kg)×restingenergyneeds(kcal/kg/day))+(weightgain(g)×energycostsofweightgain(kcal/g))

Resting energy needs were estimated at 82 kcal/kg/day, which is slightly more than required for normal growth of children aged 6 to 12 months (see Table 3.2 and 3.3 in the Food and Agricultural Organization 2001 human energy requirements report) and consistent with prior estimates of energy requirements for maintenance among young children [52]. The energy cost to add 1 g of tissue was estimated at 5kcal based on several studies cited in the Food and Agricultural Organization 2001 report (pg 31) [51].

Energy needs to support observed growth were calculated for each patient visit as kcal/day per the above formula.

For children admitted to the clinic with SAM-associated MUACs (MUAC 100mm to <115mm) and with MAM-associated MUACs (MUAC 115mm to <125mm), the smallest amount of energy that would be sufficient to achieve observed growth in 95% of visits, i.e. the 95^th^ percentile of their energy requirements, was calculated.

In secondary analyses, we compared the 95^th^ percentile of energy needs by age group (6–23 months and 24–59 months), continent (Asia and Africa), and MUAC category at admission (<115mm and 115 to <125mm). We selected the above age ranges because most children who are admitted to community-based management of acute malnutrition (CMAM) programmes are between the ages of 6–23 months, and children <24 months are the most vulnerable to episodes of acute malnutrition. The comparison by MUAC category at admission could only be made over ranges of MUAC that these children had in common (110mm to <125mm) since, in general, patients with eligible outcomes who were admitted with MUAC of 115mm or above did not generally return to the clinic with a MUAC below 110mm during treatment. The 95^th^ percentile was calculated over 5mm MUAC categories (such as MUAC 110 to <115mm). The 95^th^ percentile of energy needs was not reported to reflect average energy needs, but the provision of energy sufficient for observed growth in 95% of patient visits in the category.

Having developed a simplified protocol based on the above analyses, we assessed its theoretical performance among 100 different subsamples of 1000 patient visits each (200 patients from each country). One-hundred simulations were found to provide similar results as 1000 simulations in initial testing, so 100 simulations were considered suitable to achieve stable results. Two-hundred patient visits were selected from each country so that the patients representing each country would vary in every subsample (Yemen had the fewest cases at 294 patients). In each of the 100 simulated trials of the protocol, we recorded performance measures among patient visits with SAM-associated MUACs (100mm to <115mm) and MAM-associated MUACS (115mm to <125mm) separately. For patient visits with SAM-associated MUACs and (separately) with MAM-associated MUACs, we estimated the median percentage of energy needs covered by the protocol. For patient visits with SAM-associated MUACs, we have reported the mean percentage of visits among the 100 subsamples in which at least 100% of energy needs to support observed growth would be provided. For patient visits with MAM-associated MUACs, we have reported the mean percentage of visits in which at least 50% of energy needs to support observed growth would be provided.

We compared how the proposed protocol would compare in terms of energy provision to other protocols: namely comparing average energy provision by MUAC from Golden’s minimum (135 kcal/kg/day), intermediate (150 kcal/kg/day), and standard (170 kcal/kg/day) protocols [53]; the Sierra Leone protocol (175 kcal/kg/day if MUAC<115mm, 75 kcal/kg/day if MUAC 115mm to <125mm) [18]; and the National Guideline for Integrated Management of Acute Malnutrition for Kenya (or “Kenya protocol”, 200 kcal/kg/day if admitted to SAM treatment program, 500 kcal/day if admitted to MAM treatment program) [47]. The Golden protocols call for complete energy needs to be provided through therapeutic care and provides minimum and intermediate options with lower energy provisions for resource-constrained settings. The Kenya protocol provides complete energy needs to patients with SAM until they are discharged as recovered. Like the proposed protocol, the Sierra Leone protocol and Kenya protocol provide only supplemental energy to children with MAM. The Sierra Leone protocol also uses MUAC as the primary anthropometric admission criterion, with oedema also indicating SAM treatment should be provided.

### Ethical approval

Stage 1 of the ComPAS study was approved by the London School of Hygiene and Tropical Medicine ethics committee (reference number 11826).

## Results

### Participants

**Fig 1:**
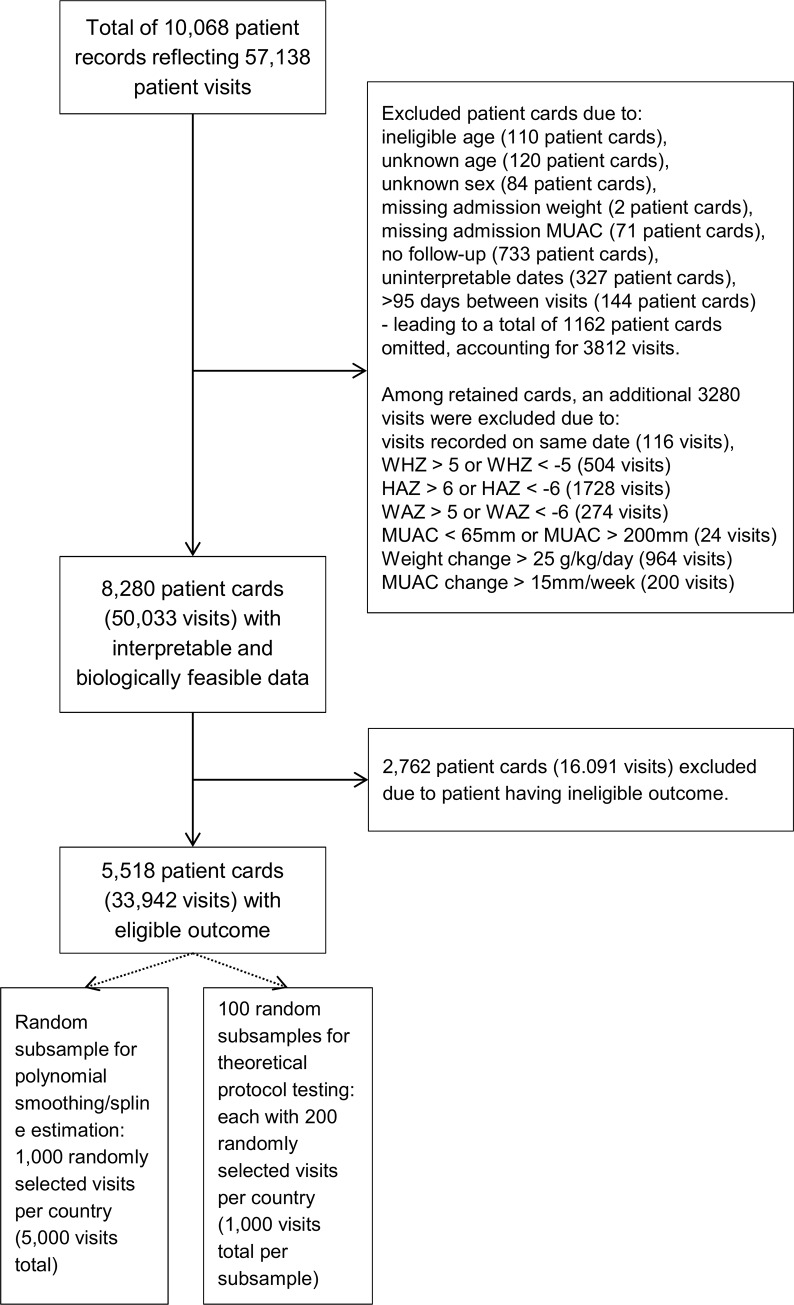
Study flow chart.

A total of 10,068 patient record cards from TFP and SFP programs in Kenya, Pakistan, Chad, Yemen, and South Sudan representing 57,138 patient visits were collected (Fig 1). Of these, we excluded 1,788 records (7,105 patient visits) from analysis due to unusable data or biologically implausible data. Of the 8,280 patient cards with usable and biologically plausible data, 5,518 patient cards (representing 33,942 visits) came from patients who ultimately had eligible outcomes.

### Descriptive data

In all countries but South Sudan, there were more female patients than male patients (Table 1). In all countries, age was frequently rounded to years rather than months, resulting in imprecise age data. Most patients were under 2 years old, with 29% of patients age 6–11 months, and 40% age 7–23 months. In Pakistan, nearly half of patients had no height data to assess for stunting. Data on presence of oedema were almost always missing or indicative of no oedema; if all non-zero, non-missing oedema values indicated presence of oedema, 11 patients would have been recorded as having oedema, almost all in South Sudan. In South Sudan, all data were from TFP facilities treating SAM; in other countries, 67–82% of patient registration cards came from SFP facilities treating MAM.

**Table 1:** Sample description.

	Kenya	Pakistan	Chad	Yemen	S. Sudan	Total
**Total number of patient cards**	439	1952	1422	483	1222	5518
… representing X visits	2701	9052	10085	2916	9188	33942
**Age group**, n (%)						
6–11 months	146 (33%)	493 (25%)	548 (39%)	113 (23%)	326 (27%)	1626 (29%)
12–23 months	135 (31%)	813 (42%)	576 (41%)	128 (27%)	544 (45%)	2196 (40%)
24–35 months	77 (18%)	400 (20%)	211 (15%)	69 (14%)	248 (20%)	1005 (18%)
36–59 months	81 (18%)	246 (13%)	87 (6%)	173 (36%)	104 (9%)	691 (13%)
**Sex**, n (%)						
Female	226 (51%)	1104 (57%)	802 (56%)	270 (56%)	585 (48%)	2987 (54%)
Male	213 (49%)	848 (43%)	620 (44%)	213 (44%)	637 (52%)	2531 (46%)
**Stunting at admission**, n (%)						
HAZ<-2	206 (47%)	876 (45%)	989 (70%)	201 (42%)	484 (40%)	2756 (50%)
HAZ≥-2	226 (51%)	370 (19%)	429 (30%)	281 (58%)	731 (60%)	2037 (37%)
Missing height	7 (2%)	706 (36%)	4 (<1%)	1 (<1%)	7 (1%)	725 (13%)
**Admitting facility**, n (%)						
TFP	121 (28%)	276 (14%)	386 (27%)	116 (24%)	1222 (100%)	2121 (38%)
SFP	318 (72%)	1676 (86%)	1036 (73%)	367 (76%)	N/A	3397 (62%)
**Oedema**, n (%)						
Oedema = 0	239 (54%)	1943 (>99%)	1421 (>99%)	483 (100%)	3 (<1%)	4089 (74%)
Missing	200 (46%)	8 (<1%)	1 (<1%)	0 (0%)	1209 (99%)	1418 (26%)
Oedema = 1,2,or 3	0 (0%)	1 (<1%)	0 (0%)	0 (0%)	10 (1%)	11 (<1%)

### MUAC and weight changes

**Fig 2:**
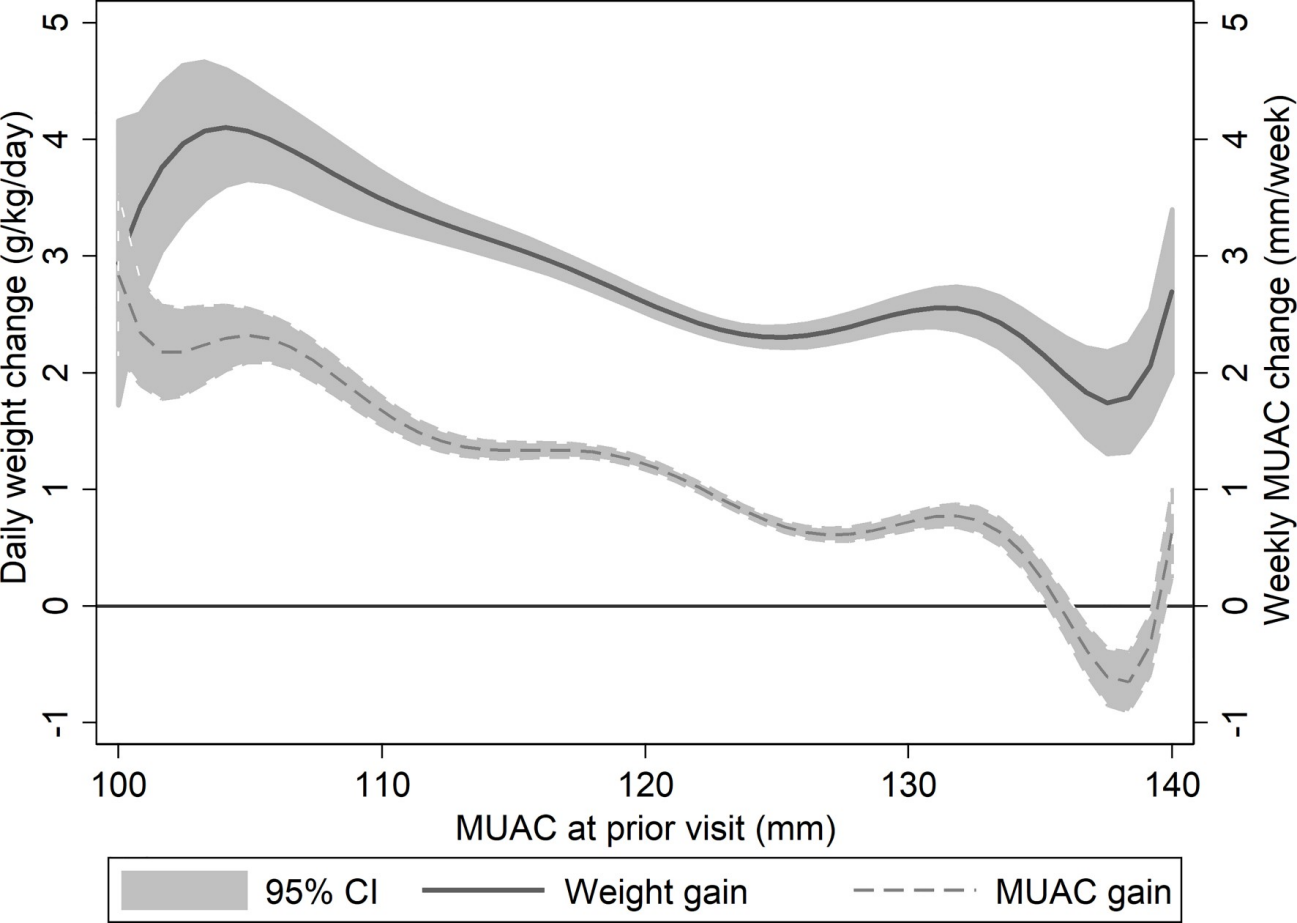
Average daily proportional weight change (solid line) and weekly MUAC change (dashed line) vs MUAC at prior visit.

Weekly MUAC change showed similar trends as proportional weight gain when assessed by MUAC (Fig 2) and were not significantly different when their values were normalized between 0 and 1 and their differences assessed for a non-zero linear trend in 100 simulations. For MUAC 100mm to <125mm, slope ranged from -0.003 to 0.002 with mean of -0.0002, with the F-test non-significant in 86% of simulations. For MUAC 125mm to 140mm, slope ranged from -0.006 to 0.001 with mean of -0.002, with the F-test non-significant in 54% of simulations (i.e., MUAC gain was slightly slower than proportional weight gain at higher MUACs in nearly half of simulations with MUAC 125mm to 140mm). Both MUAC gain and proportional weight gain were highest at lower MUACs, and declined to lower levels of growth at higher MUACs. Specifically, mean weekly MUAC change went from approximately 2mm/week at the lowest MUACs (100 to <110mm) to 1mm/week among those with MUAC 120mm to <125mm. Similarly, mean proportional weight gain declined from approximately 3.9g/kg/day to 2.4g/kg/day across the same MUAC values. An increase of 1mm MUAC was associated with a decrease in weekly MUAC growth of 0.06mm (F-test p-value<0.001) and a decrease in daily proportional weight gain of 0.05g/kg/day (F-test p-value<0.001), indicating that recovering children with higher MUACs grew more slowly according to these measures than children with lower MUACs.

### Energy needs

**Fig 3:**
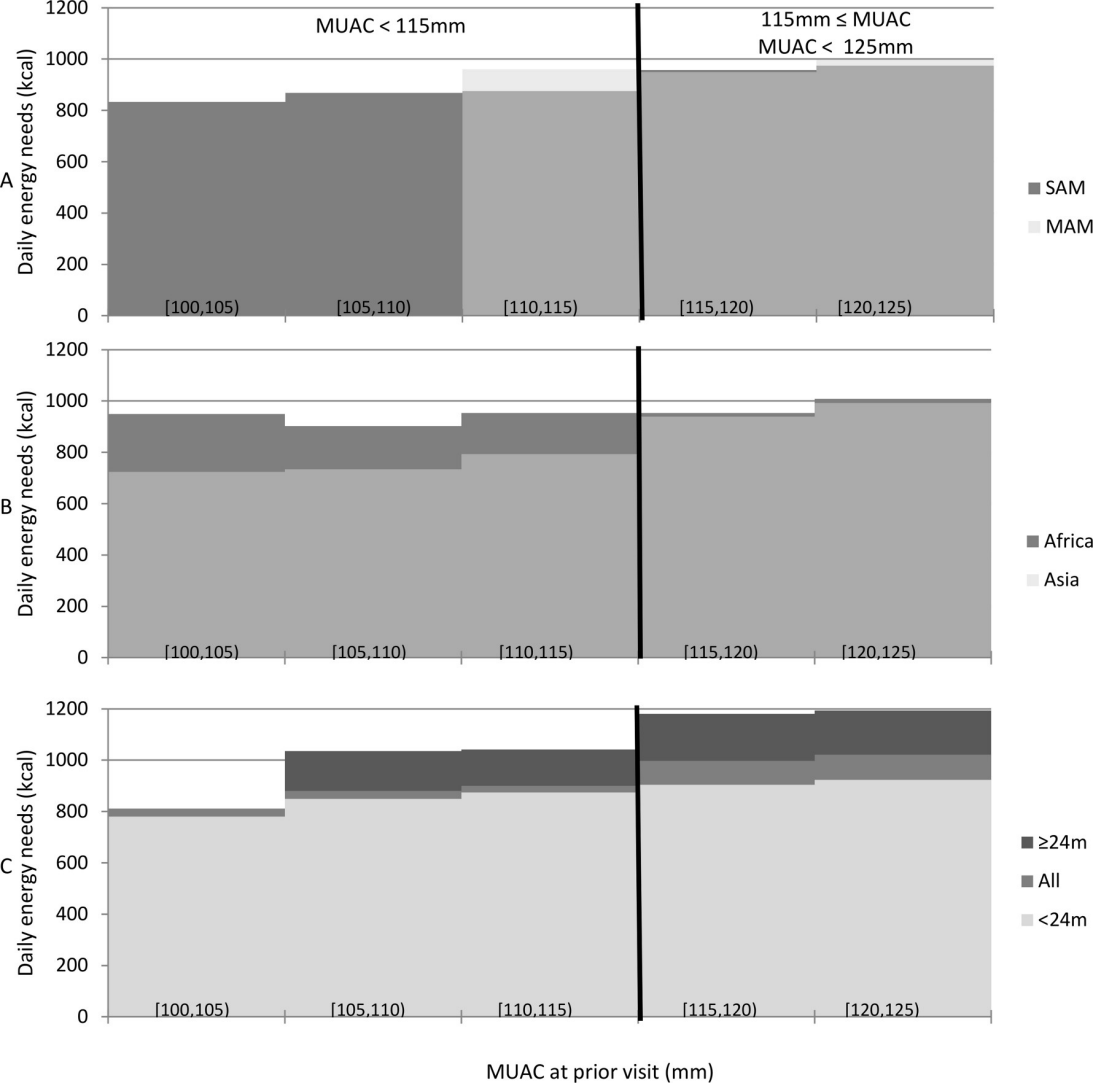
Estimated daily energy (kcal) that would provide 95% of patient visits in the specified MUAC category with energy needs for observed growth. Comparisons are made by: (A) MUAC category at admission, (B) continent, (C) and age group of 6–23 months (<24m) or 24–59 months (≥24m).

Energy need calculations indicated that 1,000 kilocalories per day were sufficient or more than sufficient in 95% of patient visits to support the growth observed among children who ultimately achieved eligible recovery outcomes (Fig 3). However, we estimated that older children (over 24 months) with higher MAM-associated MUACs (MUAC 115mm to <125mm) needed 1200kcal per day to cover 100% of energy needs to support observed growth in 95% of visits. Children over the age of 24 months made up one-fifth of the database (Table 1) and, of these, nearly all had MUACs above 115mm throughout treatment. While we estimated that most of these visits (83%) needed 1,000 kilocalories or fewer to meet their daily energy needs, this fell short of the 95% goal set for this protocol.

We noted differences in the 95th percentile of daily energy needs among children with MUAC < 115mm MUACs in Asia versus Africa, with children in African countries needing 960kcal/day versus 810kcal/day in Asia (Fig 3); however, the difference was smaller among children with MUACs 115mm to <125mm in these two regions (approximately 1050kcal/day in Africa and 995kcal/day in Asia). These results are consistent with other studies indicating that children in Asia with SAM are different from those in Africa, with a comparatively high proportion of recovery and low mortality even in the absence of intensive treatment [54].

Comparing children admitted with SAM-associated MUACs (100mm to <115mm) to those admitted with MAM-associated MUACs (115mm to <125mm), estimated energy needs did not differ greatly. The 95th percentile of daily energy needs among children admitted with MAM-associated MUACs was estimated to be 1020 kcal/day; among children admitted with SAM-associated MUACs, 960 kcal/day.

### Performance of a simplified, combined protocol

From the above, it appears that most children’s energy needs for recovery from acute malnutrition would be covered by a protocol providing 1000 kcal/day for children with SAM, whose MUAC is 100mm to <115mm and 500kcal/day for children with MAM, whose MUAC is 115mm to <125mm. That would be equivalent to two sachets per day of RUTF for children with SAM-associated MUACs, aiming to provide 100% of daily energy needs; and equivalent to one sachet per day of RUTF for children with MAM-associated MUACs and aiming to provide 50% of daily energy needs, the remainder coming from home foods [27].

**Fig 4:**
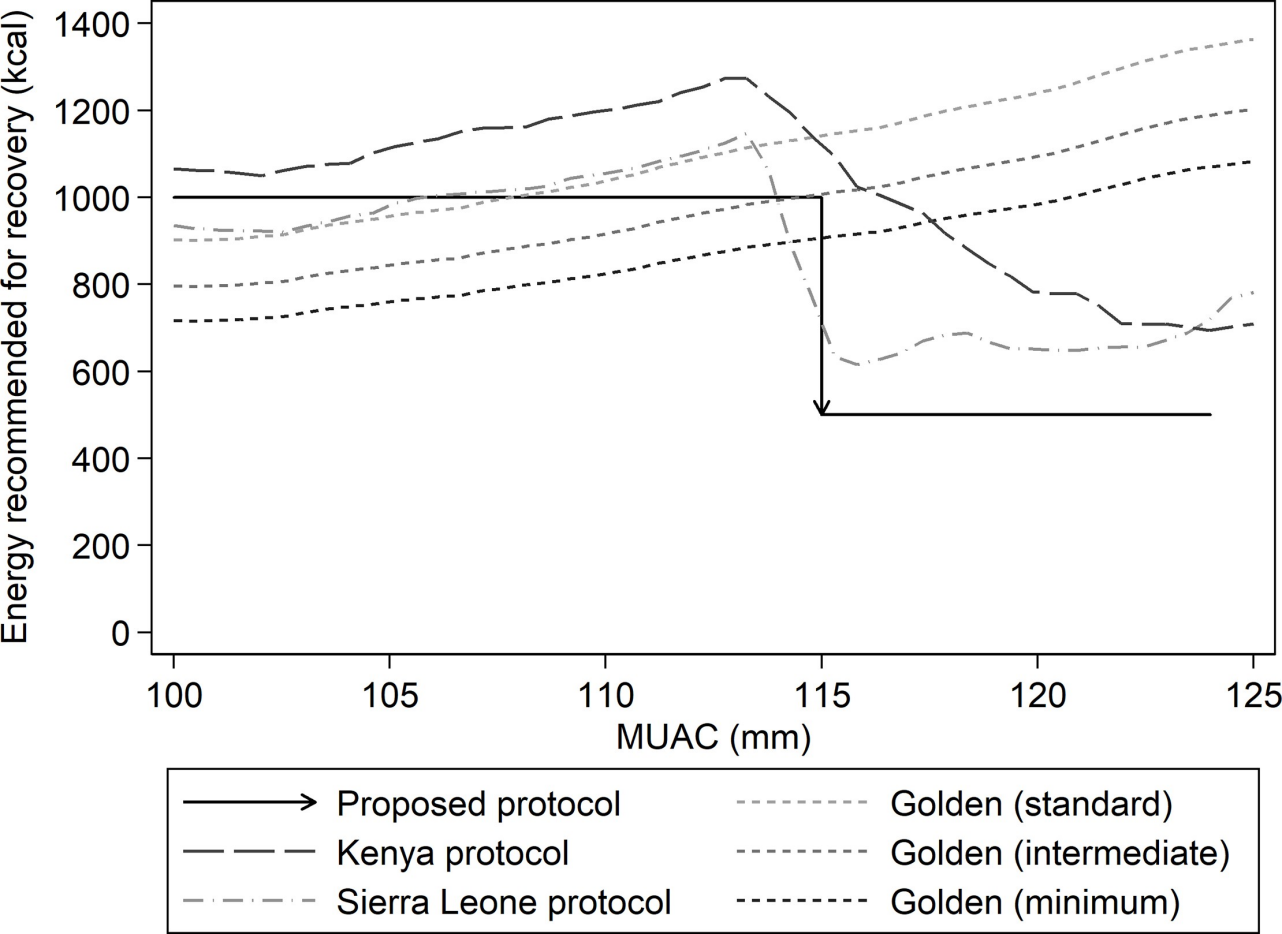
Comparison of mean energy provided by each of five protocols: The protocol proposed herein, Golden’s minimum, intermediate, and standard protocols, the Sierra Leone protocol, and the Kenya protocol.

Fig 4 shows how the proposed protocol would compare in terms of energy provision to other protocols. For lower MUACs (100mm to <115mm), the proposed protocol provides more calories than Golden’s minimum and intermediate protocols, similar kilocalories as Golden’s standard and the Sierra Leone protocol, and less than the Kenya protocol. At higher MUACs (115mm to <125mm), the proposed protocol provides approximately 100–800 fewer kilocalories per day than other protocols.

Table 2 shows the proportions of clinic visits in which the proposed protocol would provide at least 100% of estimated energy needs for children with MUAC < 115mm and at least 50% of estimated energy needs for children with MUAC 115mm to <125mm in 100 simulated trials using subsamples of 1000 visits (200 from each country) per trial. Overall, we found the protocol to be adequate for both male and female patients presenting with MAM and SAM, for children in Africa and Asia, and in each of the five countries. There were however subgroups whose energy needs were met in less than 95% of visits: older children (ages 24 to 59 months) and larger children (weights ≥ 8.0 kg). Among the older children with the lowest MUACs (age 24 to 59 months, MUAC < 115mm), total energy needs were met on an average of 90% of visits (range across simulated trials 72%-100%). Among the largest children with the lowest MUACs, their total energy needs were met on an average of 83% of visits (range 50%-100%). Estimates for these groups were not reliable due to only 1–2% of visits coming from children fitting this description and each subsample having 2 to 29 such visits to assess. For older children with higher MUACs (115mm ≤ MUAC < 125mm), an average of 83% (range 77%-88%) of visits would have had at least 50% of energy needs met by this protocol in the simulated trials. Similarly, among visits from larger children (weight ≥ 8kgs) with higher MUACs, an average of 84% (range 79%-90%) had half or more of their energy needs covered. Children with higher MUACs who were age 24 months or older and/or weighed 8kgs or more made up about one-fifth of the 5,518 patients in the sample. In both cases, these values might be far enough from the goal of 95% to warrant special consideration for these groups in the development of a simplified protocol.

**Table 2:** Estimated energy provided by proposed protocol as percentage of energy needed in observational data using 100 trials using subsamples of 200 visits from each of five countries (1000 visits total per simulated trial).

	Visits where MUAC < 115mm				Visits where 115mm ≤ MUAC < 125mm			
Factor	Mean N (min-max)	Estimated median percentage of energy needs provided by proposed protocol	Mean percentage of visits with all energy requirements provided by protocol (Target: at least 95%)	Minimum and maximum value among subsamples	Mean N (min-max)	Estimated median percentage of energy needs provided by proposed protocol	Mean percentage of visits with half of energy requirements provided by protocol (Target: approx. 95%)	Minimum and maximum value among subsamples
Total	199 (173–225)	166%	97%	94%-100%	801 (775–827)	73%	94%	92%-95%
Sex								
Female	125 (100–148)	168%	97%	95%-100%	432 (400–471)	75%	95%	92%-97%
Male	74 (59–94)	161%	96%	89%-100%	369 (331–401)	71%	92%	88%-95%
Age (in months)								
6 to 11	115 (92–138)	175%	98%	95%-100%	291 (265–335)	83%	98%	97%-100%
12 to 23	67 (49–89)	159%	96%	87%-100%	315 (270–349)	72%	96%	93%-98%
24 to 59	17 (8–29)	133%	90%	72%-100%	191 (156–220)	60%	83%	77%-88%
Weight								
3.5 to 5.9	86 (63–109)	183%	99%	95%-100%	103 (78–130)	91%	99%	97%-100%
6.0 to 7.9	106 (85–126)	157%	96%	90%-100%	470 (435–514)	77%	97%	95%-99%
8.0 to 17.5	8 (2–14)	123%	83%	50%-100%	228 (189–263)	61%	84%	79%-90%
Admission type								
SFP	See note*				453 (426–485)	74%	95%	93%-97%
TFP	195 (164–222)	166%	97%	94%-100%	347 (313–378)	72%	91%	88%-94%
Continent								
Asia	59 (41–72)	171%	99%	95%-100%	341 (328–359)	71%	94%	91%-96%
Africa	140 (116–162)	163%	96%	92%- 99%	460 (438–484)	75%	93%	90%-96%

*Among eligible patients, children who were admitted to SFP facilities seldom had MUAC below 115mm during treatment and therefore performance based on TFP vs SFP admission among children with the lowest MUACs could not be compared.

## Discussion

### Key results

We assessed how children who successfully completed a course of treatment through an TFP or SFP facility grew during their treatment, evaluating growth by the child’s MUAC at their prior clinic visit. Growth trends in MUAC mirrored those of proportional weight gain. Rate of proportional weight and MUAC gain slow at higher MUAC values. Though absolute energy requirements continue to increase as children gain weight, children with higher MUAC measurements need less supplemental energy per kilogram of body weight than those with lower MUAC measurements. According to energy estimates in this study, 1000kcal per day is hypothetically sufficient to achieve the goal of covering total energy needs 95% of the time for children with a MUAC 100mm to <115mm. For children with a MUAC 115mm-<125mm, 500kcal per day would be sufficient to supplement the family diet. The protocol met energy needs in simulated statistical tests among most subgroups (males and females, each country and continent, and both admission types), and fell slightly short of these goals among the oldest children (age 25–60 months) and the heaviest children (over 8kg).

### Interpretive considerations

This study used observational data from outpatient feeding programs in five countries in Africa and Asia, leveraging existing operational data to inform future studies and protocol development efforts. We compared MUAC and proportional weight gain as well as estimated the energy needs of patients who recovered from these programs. This information allowed us to formulate and theoretically test a simplified protocol.

Our limitations center around having retrospective data from routine clinical records rather than intervention data from a tightly controlled research setting. Records for the same children treated twice in the same program or treated for both SAM and MAM could not be connected. We were not able to account for possible confounders such as breastfeeding and socioeconomic status. We also excluded data points because of incomplete and inaccurate data collection, and used imprecise age data that were often rounded to the nearest year. As in any observational study, observed associations cannot infer causality. We calculated energy requirements to achieve observed growth using an evidence-based- but still theoretical—equation, not by controlled dosage tests. We cannot say what actual child energy intake was during the course of treatment. We also cannot say how children might have grown on different feeding regimes. Though RUTF dosages for children with SAM are relatively standard in TFPs worldwide, SFP rations vary and we did not have information on the exact type and amount of supplementary food given to children with MAM in SFP programs (possibilities include ready-to-use supplementary food and different varieties of fortified blended flours). Even if exact programme details been known, exact food/energy intake of patients was not measured and is unknown.

Another limitation arose from having very few children in some subcategories (e.g. typically fewer than 20 patients over 24 months old with SAM-associated MUACs per subsample). Therefore, we could not assess the proposed protocol’s theoretical performance for all subgroups that might be of interest to practitioners. This is not a major problem given that our programmes are representative of many others which also have most children younger than 24 months and lighter than 8kg. Care must be taken when extrapolating beyond children <24 months and <8kg.

Variations in completeness of data from the different country sources was expected. To minimize bias, the analysis was based on data that were most consistently available from all five countries. For example, because height data were missing from nearly half the cases in Pakistan, height was not used in the primary analysis, nor were height-based values such as stunting status and weight for height z-scores (WHZ).

Limiting our sample to those children with successful treatment outcomes allowed us to determine a theoretically sufficient amount of energy that would support observed growth in almost all cases represented in this sample. This analysis does not address how those children came to achieve that growth nor how children who did not successfully recover fared.

Finally, growth curves do not average the courses of individual children, but how much children tended to grow between visits given their MUAC at their prior clinic visit.

### Generalisability

These data represented growth among children age 6–59 months in standard treatment programs in five countries in Africa and Asia. The data represent very different operational contexts. The age and anthropometric characteristics of the children included in this study reflect the reality of admissions in CMAM programs globally. However, the results should not be extended to groups for which we did not have adequate data to assess, such as older and larger children with SAM and children with very low (<100mm) MUAC.

### Interpretation

This study considered the rate of weight and MUAC gain and energy needs of children with acute malnutrition as defined by MUAC status. We found that 1000 kcal/day (equivalent to two RUTF sachets per day) should meet the total energy requirements of children with a MUAC of 100mm to <115mm more than 95% of the time; 500kcal (equivalent to one RUTF sachet per day) meets half the energy requirements of children with a MUAC 115 to <125mm 95% of the time. Even among the subgroups of older and larger children that did not have these goals met 95% of the time, the protocol was estimated to meet their needs approximately 83% of the time. This protocol remains in line with globally accepted practice in which children recovering from SAM receive enough therapeutic food to cover their total energy needs, and children with MAM receive a food supplement to complement their family diet [14,27]. Most SFP protocols currently provide 500-550kcal/day of ready-to-use supplementary food (RUSF). Similarly, recently published studies indicate that admitting children with a MUAC <125mm leaves very few high risk children untreated [20,21,55].

The combined protocol developed through this study provides a novel MUAC-based protocol to test in operational and clinical trials. The combined protocol was recently tested in a cluster-randomized controlled trial in Kenya and South Sudan, with results of the trial expected in early 2020 [43].

### Other research

These results are consistent with and bolster other global child malnutrition studies [23,56]. There is increasing evidence that MUAC-based admission and discharge criteria are effective at improving efficiency and care outcomes [20,57,58]. Several extant protocols that provide supplementary foods to families of children with MAM rather than provide all energy needs with therapeutic foods have been found to be successful [18,47].

Answering the questions of whether a single protocol for the management of MAM and SAM will increase cost-effectiveness and access to treatment are among the objectives of the ComPAS stage 2 randomized controlled trial (the results of which currently under review). Since the conclusion of the ComPAS stage 2 trial, several operational studies and additional RCT’s have begun evaluating these questions as well [42,59–62]. The premise is that a single protocol will result in increased coverage of MAM cases before these children deteriorate into SAM. MAM treatment is less costly for a number of reasons: less frequent visits (bi-weekly instead of weekly), less RUTF (one sachet per day), and minimal medical treatments. In this way, we hypothesize that the increase in cost by expanding treatment to MAM will be balanced by the reduction in the more costly treatment of SAM. Additionally, an important aspect of the protocol tested in ComPAS Stage 2 is the optimized dosage for SAM children. The optimized dosage provides two sachets of RUTF per day for all children less than 115mm. In the standard protocol currently used in most countries, the dosage of RUTF is based on weight and ranges between two to five sachets per day. Therefore, an additional aspect of cost savings is not just the reduction in SAM cases, but the reduced dosage of RUTF used for SAM. We will need to see this play out in operational studies in multiple countries to assess the practical implications, and that is what the UN and non-governmental organizations (NGOs) are doing now [63].

Future research can expand on these findings by powering samples to estimate energy needs of smaller subgroups of children with acute malnutrition such as those with oedema and older and larger children with SAM. Primary data collection can be used to obtain more precise data regarding age of children for more granular analysis by age. Additionally, growth differences between children with similar MUACs but in TFP versus SFP clinics can be explored.

## Conclusion

Using data from several large therapeutic feeding programs, we found MUAC to be a good proxy for proportional weight gain, demonstrating similar growth pattern changes. Using observed growth, we estimated that 1000 kcal of energy per day is sufficient for recovery of children with uncomplicated acute malnutrition. This suggests that a simplified protocol of two RUTF packets per day for children with MUAC 100mm to <115mm and one RUTF packet per day for children with MUAC 115mm to <125mm is an acceptable therapeutic approach to uncomplicated acute malnutrition. Since most children in our sample were young, we can be most confident or our results for children aged <2 years. Further research should focus on needs of older children. In particular, intervention trials are needed for testing this protocol and other approaches.
